# Establishment and validation of a clinical prediction model for predicting early postpartum pelvic floor muscle weakness among primiparous women after vaginal delivery: a retrospective study

**DOI:** 10.3389/fmed.2025.1605662

**Published:** 2025-09-24

**Authors:** Huan Dong, Xiaolei Chi, Ye Liu, Wenjuan Liu, Xinliang Chen, Xianjing Wang, Ping Liu

**Affiliations:** 1 The International Peace Maternity and Child Health Hospital, School of Medicine, Shanghai Jiao Tong University, Shanghai, China; 2 Shanghai Key Laboratory of Embryo Original Diseases, Shanghai, China

**Keywords:** primiparous women, vaginal delivery, pelvic floor muscle weakness (PFMW), clinical prediction model, early postpartum period

## Abstract

**Background:**

Pelvic floor muscle weakness (PFMW) is a significant postpartum complication linked to pelvic floor dysfunction. PFMW impairs quality of life and requires early intervention. This study aimed to develop and validate a clinical prediction model for early postpartum PFMW in primiparous women after vaginal delivery.

**Methods:**

This retrospective cohort study was conducted at a tertiary maternity hospital in Shanghai, China. Primiparous women with vaginal deliveries (July 2021–December 2023) were enrolled. Participants were assessed for PFMW using pelvic floor surface electromyography (sEMG) via the Glazer protocol at 42–90 days postpartum. Maternal and obstetric predictors were analyzed via univariable and multivariable logistic regression to construct a nomogram. Model performance was evaluated using concordance statistics (C-statistics), calibration curves, and decision curve analysis in both the training (*n* = 2,465) and validation (*n* = 1,049) cohorts. Internal validation was performed via ten-fold cross-validation.

**Results:**

Among 3,514 enrolled women, PFMW occurred in 25.55% (898/3,514), with comparable baseline characteristics between cohorts (age, pre-pregnancy BMI; *P* > 0.05). Multivariable analysis revealed five independent predictors: maternal age (OR 1.156, 95% CI 1.116–1.999), gestational weight gain (OR 1.146, 95% CI 1.116–1.178), instrumental delivery (forceps: OR 1.904, 95% CI 1.336–2.714), prolonged second stage of labor (OR 1.026, 95% CI 1.022–1.029), and infant weight (OR 1.003, 95% CI 1.002–1.003). The nomogram demonstrated strong discrimination [C-statistic: 0.866 (95% CI 0.850–0.882) in the training cohort; 0.870 (0.819–0.903) in the validation cohort] and good calibration. Decision curve analysis confirmed the clinical utility across threshold probabilities (0–0.3).

**Conclusion:**

This study established a validated nomogram integrating maternal and obstetric factors to predict early postpartum PFMW in primiparous women after vaginal delivery. This tool may aid in the early identification of high-risk individuals, enabling targeted rehabilitation to mitigate long-term pelvic floor dysfunction.

## Introduction

Pelvic floor dysfunction (PFD) is a clinical syndrome caused by weakened pelvic floor support structures, leading to displacement and functional abnormalities of pelvic organs. Its primary manifestations include pelvic organ prolapse (POP), urinary incontinence (UI), fecal incontinence, and sexual dysfunction (1). PFD is now recognized as a “hidden epidemic”, with epidemiological data indicating that 21%−26% of women are affected by this condition (2). Although PFD is not life-threatening, it significantly impairs patients' quality of life and increases the risk of psychological issues such as anxiety and depression, making it a critical focus in global obstetrics and gynecology research. Among the numerous risk factors for PFD, pregnancy and childbirth are the most influential independent risk factors (1, 3). Previous studies have confirmed that 75% of POP cases are directly attributable to pregnancy- and childbirth-related trauma (4). Among women who undergo vaginal delivery, 25%−50% develop varying degrees of UI, fecal incontinence, or POP within 1 year postpartum (5); this phenomenon is closely linked to mechanical damage to the pelvic floor muscles (PFM) and nerves during delivery (6).

PFM includes type I slow-twitch fibers (which are responsible for static pelvic support) and type II fast-twitch fibers (which are responsible for regulating dynamic contraction). Dysfunction in PFM, which is characterized by reduced muscle tone or loss of coordinated contraction, leads to anatomical displacement and functional impairment of organs such as the bladder, uterus, and rectum, which underlies the pathophysiological mechanism of PFD (7). Current methods for assessing PFM function include visual inspection, vaginal palpation, ultrasound, magnetic resonance imaging (MRI), manometry, infrared thermography (IRT), and surface electromyography (sEMG) (8–11). However, no gold standard exists for scientific research or clinical practice, as each method has specific advantages and limitations. Among these, the Glazer protocol for pelvic floor surface electromyography, proposed by Glazer and Marinoff in 1997, has been clinically validated as a reliable and effective non-invasive method for PFM electromyographic evaluation (12, 13).

Reported risk factors for PFD include maternal and obstetric indicators such as mode of delivery (vaginal delivery poses greater risks than cesarean section), instrument-assisted delivery (e.g., forceps), parity, advanced maternal age at first delivery, high BMI, and fetal birth weight (14–19). Socioeconomic factors (low income, rural residence and physical labor) and perinatal management (e.g., absence of oxytocin use) have also been implicated in the risk profile (20, 21). Nevertheless, independent predictors of PFMW in primiparous women after vaginal delivery, and the reason remain unclear, particularly because few large-scale studies have quantified the associations between maternal-obstetric indicators and functional impairment in PFM.

This study employs the Glazer protocol to evaluate early postpartum PFM function in primiparous women following vaginal delivery. By constructing a clinical prediction model that integrates maternal characteristics and obstetric indicators, we aimed to develop a nomogram for predicting the risk of PFMW. This tool will enable early screening and targeted interventions for high-risk populations, thus providing evidence-based strategies for preventing postpartum and long-term PFD.

## Materials and methods

### Study design and population

This single-center observational study was performed at the International Peace Maternity and Child Health Hospital (IPMCH), Shanghai Jiao Tong University School of Medicine, which is designated for early postpartum primiparous women with vaginal delivery. IPMCH is one of the largest obstetric care centers in Shanghai, with 10,000~16,000 annual deliveries. All the participants officially consented both verbally and in writing to participate in the study, and the protocol was approved by the Ethics Committee of the IPMCH (No. GKLW-2023-024-01). Primiparous women who underwent vaginal delivery between July 2021and December 2023 were enrolled in the study cohort and then randomly allocated to either the training cohort or the validation cohort. The inclusion criteria were as follows: (a) primiparas after vaginal delivery; (b) singleton cephalic presentation at 34–41 weeks of gestation; (c) 42–90 days postpartum; (d) normal mental state with good cooperation during inspection. The exclusion criteria were as follows: (a) history of urinary leakage, chronic constipation, pelvic floor disorders, and uterine or pelvic floor surgery; (b) history of connective tissue disease or myasthenia gravis; (c) severe hearing impairment, intellectual disability, and severe cardiorespiratory insufficiency; (d) history of miscarriage after 16 gestational weeks; (e) abnormal postpartum recovery (including vaginal bleeding, failure of the uterus to contract into the pelvis, poor healing of a perineal laceration or lateral episiotomy); (f) presence of abdominal or internal hip muscle hypercontraction during evaluation; (g) presence of abdominal muscle sEMG of >10 μV during PFM contraction; or (h) patients who were lost to follow-up or whose clinical data was missing.

### Research methods

All the subjects were assessed for pelvic floor function at 42–90 days postpartum by measuring pelvic floor muscle sEMG signals using vaginal surface electrodes (Glazer Protocol). The maternal and obstetric-related data were retrieved from the electronic medical records of the hospital. The general demographic characteristics included maternal age, pre-pregnancy body mass index (BMI) and educational level. The baseline characteristics assessed during pregnancy included weight gain during pregnancy, diabetes (gestational/pregestational), hypertensive disorders, abnormal thyroid function and anemia (hemoglobin level of less than 11 g/dl) (22), gestational age at delivery and infant weight. The baseline characteristics during labor included premature rupture of membranes (PROM), epidural anesthesia, the first stage of labor (time from the onset of regular contractions to full cervical dilation), the second stage of labor (time from the cervix being fully dilated to complete delivery of fetus), episiotomy (routine episiotomies are mediolateral), perineal lacerations, intrapartum blood loss and mode of delivery (including spontaneous vaginal delivery, vacuum-assisted delivery and forceps delivery).

### Evaluation methods and diagnostic indicators

In this study, a neuromuscular stimulation instrument (SA9800, MLD B4, Medlander Medical Technology Inc., Nanjing, China) was used for sEMG testing, and the sEMG signal acquisition device was a custom-made vaginal metal probe (CACB04, MLD V1, Medlander Medical Technology Inc., Nanjing, China). Software analysis was performed on a MYOTRAC Inffniti system (Montreal, Canada), and the results are expressed in μV. The participants were placed in a supine position with a pear-shaped vaginal metal probe placed inside the vagina, and the electrode devices were placed on the hip adductor, gluteus and abdominal muscles to monitor unwanted muscle activation. All operations were performed by professionally trained medical staff at the Pelvic Floor Screening and Rehabilitation Center of International Peace Maternity and Child Health Hospital. Prior to the test, all the subjects were informed of the complete test procedure and were instructed on how to properly contract the PFM to avoid abdominal and internal hip muscle crosstalk. During the assessment, a visual screen with voice prompts instructed the subjects when to contract and when to relax. Professional medical staff provided personalized guidance and practice opportunities for the participants, and all participants completed the test successfully.

As per the Glazer Protocol, we divided the test into two phases: a fast-twitch muscle (type II fiber) phase and a slow-twitch muscle (type I fiber) phase. During the fast-twitch muscle evaluation phase, after short-term pelvic floor muscle contractions, the maximum (peak) values were recorded, and fast-twitch muscle function was evaluated. During the slow-twitch muscle evaluation phase, five slow and gentle PFM contractions and a sustained maximum contraction for 10 s were performed, with the reported value being the average of five measurements. This stage can be used to evaluate slow-twitch muscle strength and fast–slow muscle coordination. A maximum value of ≥40 μV was considered normal, whereas a maximum value of < 40 μV indicated a decrease in fast muscle strength during the fast-twitch muscle (type II fiber) phase. A maximum value of ≥35 μV was considered normal, whereas a maximum value of < 35 μV indicated decreased slow muscle strength in the slow-twitch muscle phase (type I fiber) (11, 13, 23). Participants with either fast-twitch or slow-twitch muscle strength decline were categorized into the pelvic floor muscle weakness (PFMW) group, while the remaining individuals were assigned to the normal control group.

## Statistical analysis

Continuous variables are presented as the mean ± standard deviation or as the median (interquartile range). Variables were compared via Student's *t* test or the Mann–Whitney *U* test, as appropriate. Categorical variables are presented as frequencies and percentages and were compared using the chi-square test (or Fisher's exact test when the expected frequency was < 5).

In the entire training cohort, univariable analysis was used to identify factors that were significantly associated with PFMW. Variables that were found to be associated with PFMW in the univariate analysis (*P* < 0.2) were subsequently added to the multivariate analysis, and backward stepwise selection was carried out with an improvement in goodness of fit measured by a decrease in the Akaike information criterion. A nomogram for PFMW likelihood was developed on the basis of the findings of the final regression analysis.

Concordance statistics (C-statistics) and 95% confidence intervals (CIs) were computed to evaluate the ability of the nomogram to identify patients who will suffer from PFMW. Furthermore, the C-statistics between the nomogram and each independent predictor were compared using the Delong test. Calibration curves were developed via 1,000 bootstrap resamples to analyze the level of agreement between the nomogram predictions and actual observations in the training cohort. Decision curve analysis was performed by estimating the net benefits at various threshold probabilities of PFMW to evaluate the clinical utility of the predictive nomogram.

Internal validation of the model's stability was carried out via cross-validation, which involved randomly dividing the training cohort's patients into ten equal samples. To create logistic regression models, nine of these samples were used, and the final sample was then given the model coefficients. The mean C-statistics for each iteration was computed after this procedure was repeated ten times. Additionally, the model was applied to a validation dataset and evaluated via C statistics, calibration, and decision curve analysis to evaluate its external validity.

R version 4.4.1 was used for statistical analyses and for graphing. All statistical analyses were two–sided tests, and *P* < 0.05 was considered statistically significant.

## Results

### General characteristics

**Figure 1:**
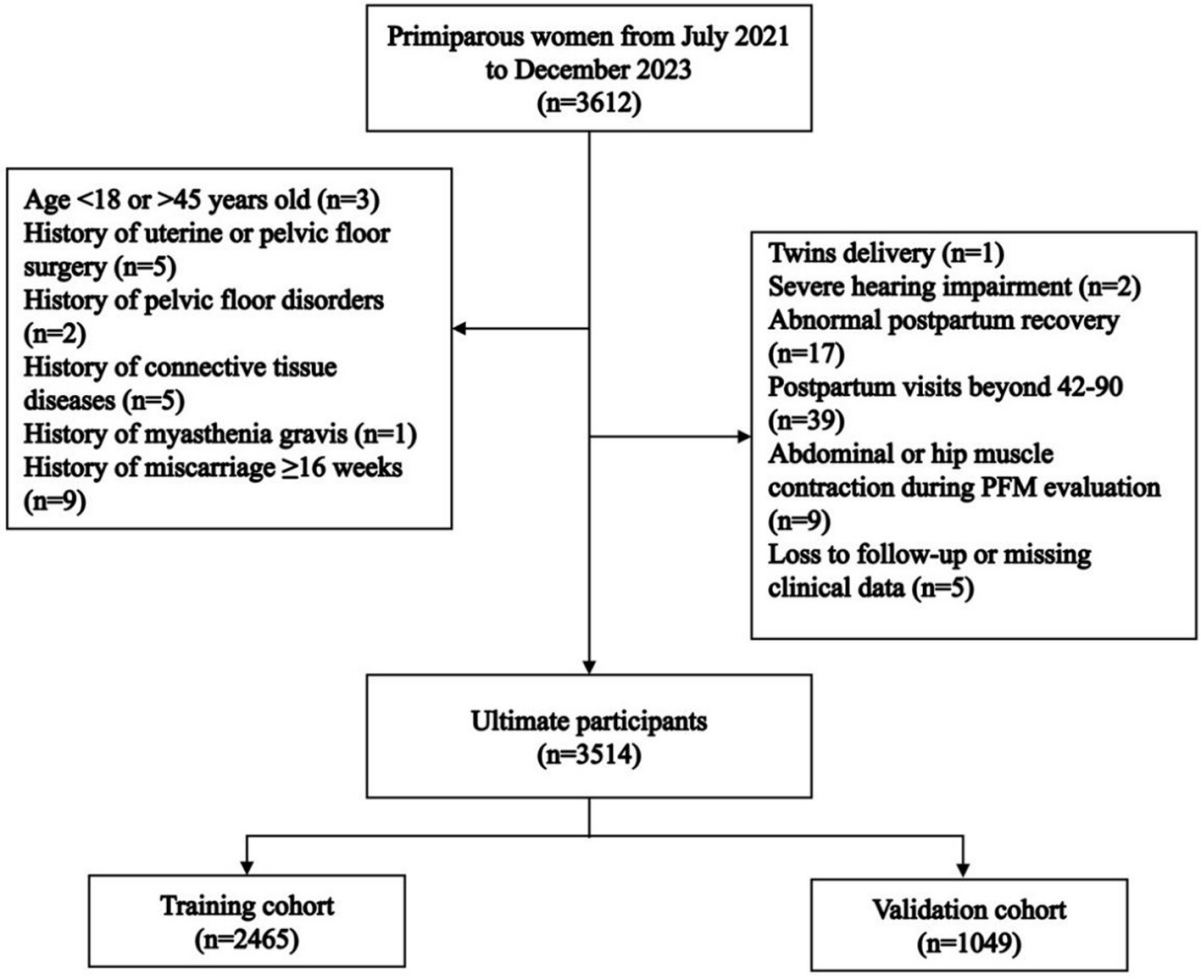
Flow chart of the study participants.

A total of 3,612 primiparous women who delivered vaginally during the study period were recruited. Among these, 98 women were excluded. Ultimately, 3,514 women were enrolled in this study, with 2,465 in the training cohort and 1,049 in the validation cohort (Figure 1). There were no statistically significant differences in age [30 (28, 31) vs. 30 (28, 32), *P* = 0.972] or pre-pregnancy BMI [20.31 (18.93, 22.03) vs. 20.31 (19.03, 22.02), *P* = 0.941] between the training and validation cohorts (Table 1). PFMW occurred in 898 (25.55%) patients overall, including 624 (25.31%) and 274 (26.12%) in the training cohort and validation cohort, respectively (Table 1).

**Table 1:** Demographic, pregnancy and delivery characteristics of the training cohort and validation cohort.

	**Training cohort**, ***M*** **(P25, P75)/*****n*** **(%)**					**Validation cohort **, ***M*** **(P25, P75)/*****n*** **(%)/**$\bar{x}\;$±**s**					**^†^ *P***
**Variables**	**Total**	**Non-PFMW**	**PFMW**	***t*****/**χ^2^**/*****Z***	^*^ ***P***	**Total**	**Non-PFMW**	**PFMW**	***t*****/**χ^2^**/*****Z***	^*^ ***P***	
	**(*****n*** = **2,465)**	**(*****n*** = **1,841)**	**(*****n*** = **624)**			**(*****n*** = **1,049)**	**(*****n*** = **775)**	**(*****n*** = **274)**			
Age, year	30 (28, 31)	29 (28, 30)	30 (29, 32)	77.704	< 0.001	30 (28, 32)	28 (27, 31)	30 (28, 31)	8.156	0.004	0.972
Pre-pregnancy BMI, kg/m^2^	20.31 (18.93, 22.03)	20.34 (18.96, 21.98)	20.27 (18.89, 22.11)	0.116	0.734	20.31 (19.03, 22.02)	20.17 (19.05, 22.04)	20.45 (19.01, 21.78)	0.174	0.570	0.941
Weight gain in pregnancy, kg	13.7 (10.9, 16.3)	13.1 (10.2, 15.8)	15.2 (12.6, 18.2)	150.112	< 0.001	13.8 (10.9, 16.7)	13.9 (11.15, 16.75)	13.55 (10, 16.62)	2.251	0.133	0.271
**Education**											
Junior high school or low	78 (3.16)	57 (3.1)	21 (3.37)	6.549	0.088	37 (3.53)	26 (3.35)	11 (4.01)	5.873	0.118	0.330
High school	353 (14.32)	245 (13.31)	108 (17.31)			158 (15.06)	109 (14.06)	49 (17.88)			
Junior college or university	1316 (53.39)	1000 (54.32)	316 (50.64)			580 (55.29)	424 (54.71)	156 (56.93)			
Graduate or above	718 (29.13)	539 (29.28)	179 (28.69)			274 (26.12)	216 (27.87)	58 (21.17)			
**Diabetes (gestational/pregestational)**											
No	2140 (86.82)	1597 (86.75)	543 (87.02)	0.011	0.916	884 (84.27)	645 (83.23)	239 (87.23)	2.152	0.142	0.052
Yes	325 (13.18)	244 (13.25)	81 (12.98)			165 (15.73)	130 (16.77)	35 (12.77)			
**Hypertensive disorders**											
No	2341 (94.97)	1751 (95.11)	590 (94.55)	0.200	0.655	998 (95.14)	741 (95.61)	257 (93.8)	1.079	0.299	0.900
Yes	124 (5.03)	90 (4.89)	34 (5.45)			51 (4.86)	34 (4.39)	17 (6.2)			
**PROM**											
No	1737 (70.47)	1287 (69.91)	450 (72.12)	0.988	0.320	719 (68.54)	529 (68.26)	190 (69.34)	0.066	0.797	0.272
Yes	728 (29.53)	554 (30.09)	174 (27.88)			330 (31.46)	246 (31.74)	84 (30.66)			
**Anemic**											
No	1950 (79.11)	1458 (79.2)	492 (78.85)	0.017	0.897	816 (77.79)	608 (78.45)	208 (75.91)	0.616	0.433	0.407
Yes	515 (20.89)	383 (20.8)	132 (21.15)			233 (22.21)	167 (21.55)	66 (24.09)			
**Abnormal thyroid function**											
No	2294 (93.06)	1721 (93.48)	573 (91.83)	1.729	0.189	969 (92.37)	719 (92.77)	250 (91.24)	0.475	0.490	0.513
Yes	171 (6.94)	120 (6.52)	51 (8.17)			80 (7.63)	56 (7.23)	24 (8.76)			
Gestational age, week	39 (38, 40)	39 (38, 40)	39 (38, 40)	12.806	< 0.001	39 (38, 40)	39 (38, 40)	39 (38, 40)	1.499	0.221	0.048
**Mode of delivery**											
Spontaneous vaginal delivery	2164 (87.79)	1662 (90.28)	502 (80.45)	43.337	< 0.001	905 (86.27)	682 (88)	223 (81.39)	Fisher	0.015	0.170
Vacuum-assisted delivery	23 (0.93)	16 (0.87)	7 (1.12)			6 (0.57)	5 (0.65)	1 (0.36)			
Forceps delivery	278 (11.28)	163 (8.85)	115 (18.43)			138 (13.16)	88 (11.35)	50 (18.25)			
**Epidural anesthesia**											
No	705 (28.6)	524 (28.46)	181 (29.01)	0.043	0.835	305 (29.08)	217 (28)	88 (32.12)	1.470	0.225	0.807
Yes	1760 (71.4)	1317 (71.54)	443 (70.99)			744 (70.92)	558 (72)	186 (67.88)			
**Perineal lacerations**											
None	36 (1.46)	31 (1.68)	5 (0.8)	22.455	< 0.001	10 (0.95)	7 (0.9)	3 (1.09)	Fisher	0.260	0.270
I	819 (33.23)	648 (35.2)	171 (27.4)			322 (30.7)	247 (31.87)	75 (27.37)			
II	921 (37.36)	686 (37.26)	235 (37.66)			410 (39.08)	306 (39.48)	104 (37.96)			
III, IV and episiotomy	689 (27.95)	476 (25.86)	213 (34.13)			307 (29.27)	215 (27.74)	92 (33.58)			
**Episiotomy**											
No	1803 (73.14)	1365 (74.14)	438 (70.19)	3.507	0.061	742 (70.73)	560 (72.26)	182 (66.42)	3.053	0.081	0.155
Yes	662 (26.86)	476 (25.86)	186 (29.81)			307 (29.27)	215 (27.74)	92 (33.58)			
First stage of labor, h	6.25 (4.17, 9)	6.17 (4, 9)	6.5 (4.5, 9)	2.302	0.129	6.02 (4, 9)	6 (4, 9)	6.5 (4, 9)	0.906	0.341	0.189
Second stage of labor, min	42 (26, 67)	36 (22, 56)	62.5 (41, 98)	334.744	< 0.001	43 (27, 69)	42 (26, 67)	45 (27, 72)	2.033	0.154	0.227
Intrapartum blood loss, ml	320 (260, 370)	315 (260, 370)	325 (270, 376.25)	5.759	0.016	320 (265, 370)	315 (265, 362.5)	335 (270, 388.75)	6.156	0.013	0.516
Infant weight, g	3,260 (3,035, 3,500)	3,190 (2,960, 3,410)	3,480 (3,265, 3,825)	374.410	< 0.001	3,270.78 ± 367.72	3,258.13 ± 370.49	3,306.57 ± 358.04	3.521	0.061	0.649

PFMW, pelvic floor muscle weakness; BMI, body mass index; PROM, premature rupture of the membranes of the fetus.

^*^P value for the difference between women with PFMW and non-PFMW;^†^*P* value for the training cohort vs. the validation cohort for total characteristics.

### Selected factors for the model

Univariable analysis revealed that age, weight gain during pregnancy, gestational age, mode of delivery, perineal lacerations, second stage of labor, intrapartum blood loss and infant weight were significantly associated with PFMW and were thus entered into the multivariable logistic regression analysis. The multivariable analyses revealed that the occurrence of PFMW was significantly correlated with age, weight gain during pregnancy, mode of delivery, second stage of labor and infant weight (*P* < 0.001). Therefore, the above five factors were selected for the final model (Table 2).

**Table 2:** Multivariable analysis of the training cohort.

**Variables**	**Multivariate analysis**			**Selected factors for model**		
	**OR**	**95% CI**	***P***	**OR**	**95% CI**	***P***
Age, year	1.158	1.117–1.201	< 0.001	1.156	1.116–1.199	< 0.001
Weight gain in pregnancy, kg	1.152	1.120–1.184	< 0.001	1.146	1.116–1.178	< 0.001
Gestational age, week	0.942	0.843–1.053	0.293			
Mode of delivery			0.001			< 0.001
**Spontaneous vaginal delivery**						
Vacuum-assisted delivery	1.688	0.530–5.376	0.376	1.499	0.491–4.580	0.478
Forceps delivery	2.282	1.455–3.578	< 0.001	1.904	1.336–2.714	< 0.001
Perineal lacerations			0.737			
**None**						
I	1.814	0.482–6.824	0.379			
II	1.823	0.486–6.843	0.374			
III, IV and episiotomy	1.612	0.420–6.187	0.486			
Second stage of labor, min	1.026	1.022–1.030	< 0.001	1.026	1.022–1.029	< 0.001
Intrapartum blood loss, ml	0.999	0.998–1.000	0.137			
Infant weight, g	1.003	1.002–1.003	< 0.001	1.003	1.002–1.003	< 0.001

OR, odds ratio; CI, confidence interval.

### Risk prediction nomogram establishment

**Figure 2:**
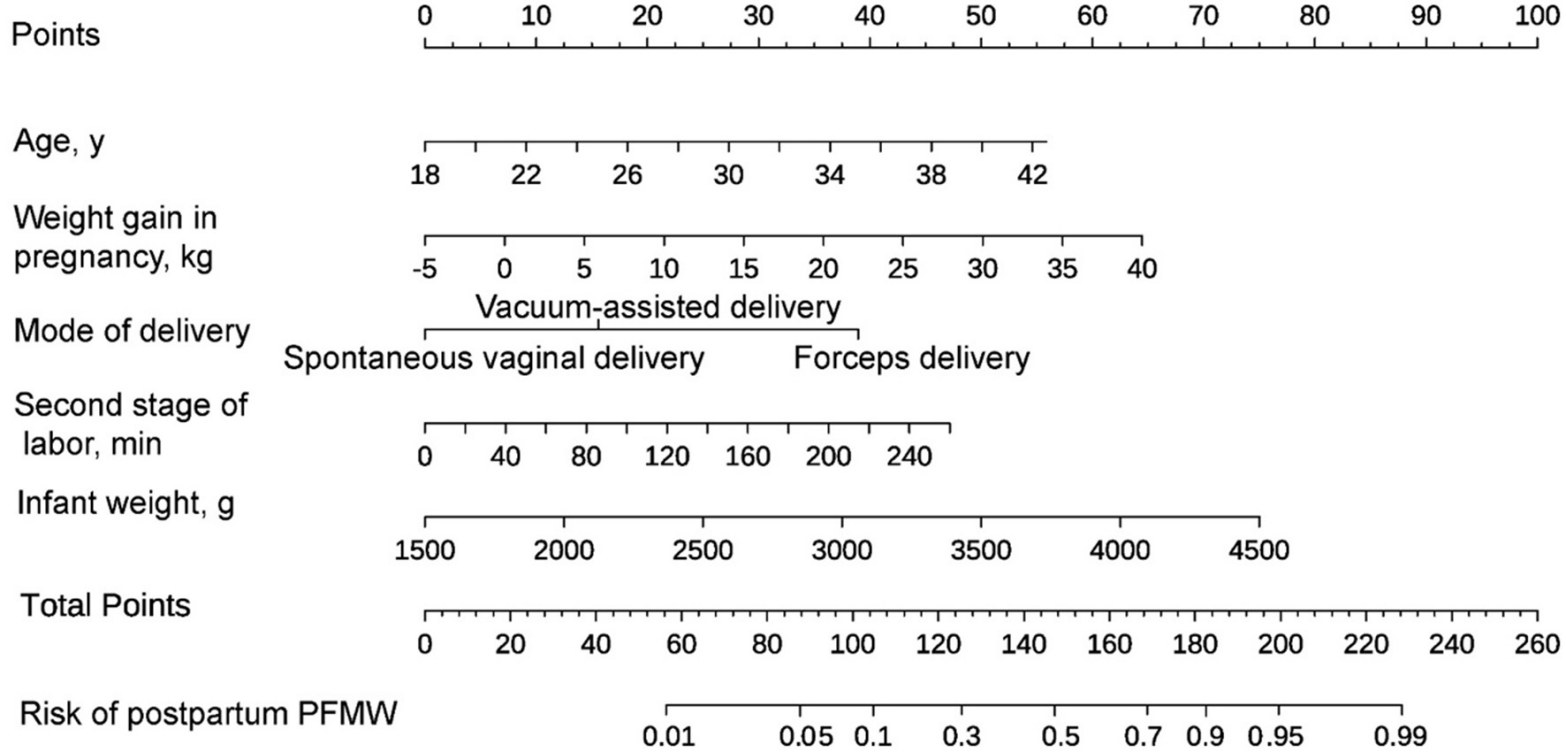
A nomogram for predicting postpartum pelvic floor muscle weakness (PFMW) in primiparous women with vaginal delivery. On the axis of the point scale, the value of each variable was assigned a score. The probability of postpartum PFMW can be determined by adding each individual score and by projecting the result to the lower total point scale.

The final regression analysis was used to create a nomogram for predicting PFMW. Age, weight gain during pregnancy, mode of delivery, second stage of labor and infant weight were used to obtain a total score. Each of these variables' values received a score on the axis of a point scale. Each individual score can be readily summed to obtain a total score, and by extrapolating the total score to the entire point scale, the likelihood of PFMW can be calculated (Figure 2).

### Performance of the nomogram

**Figure 3:**
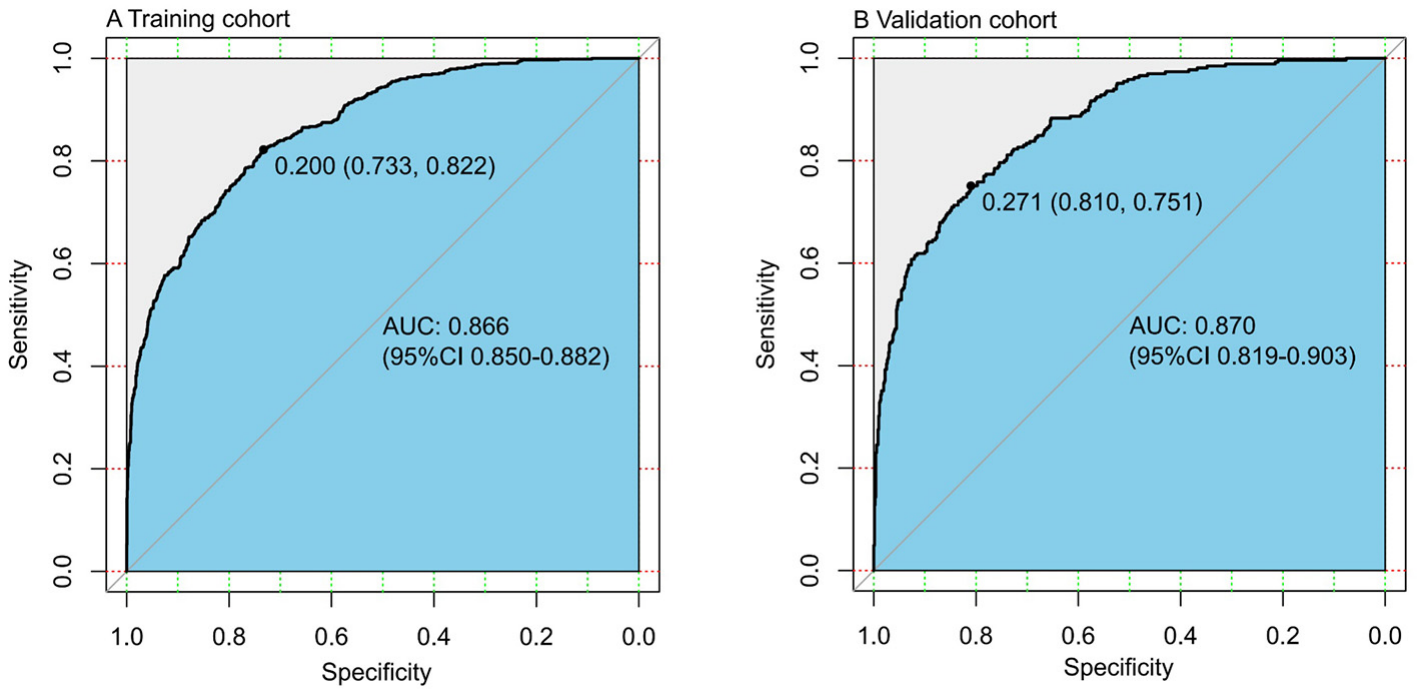
Receiver operating characteristic (ROC) curve. AUC, area under the ROC curve, equal to the C-statistic value. **(A)** Training cohort. **(B)** Validation cohort.

**Figure 4:**
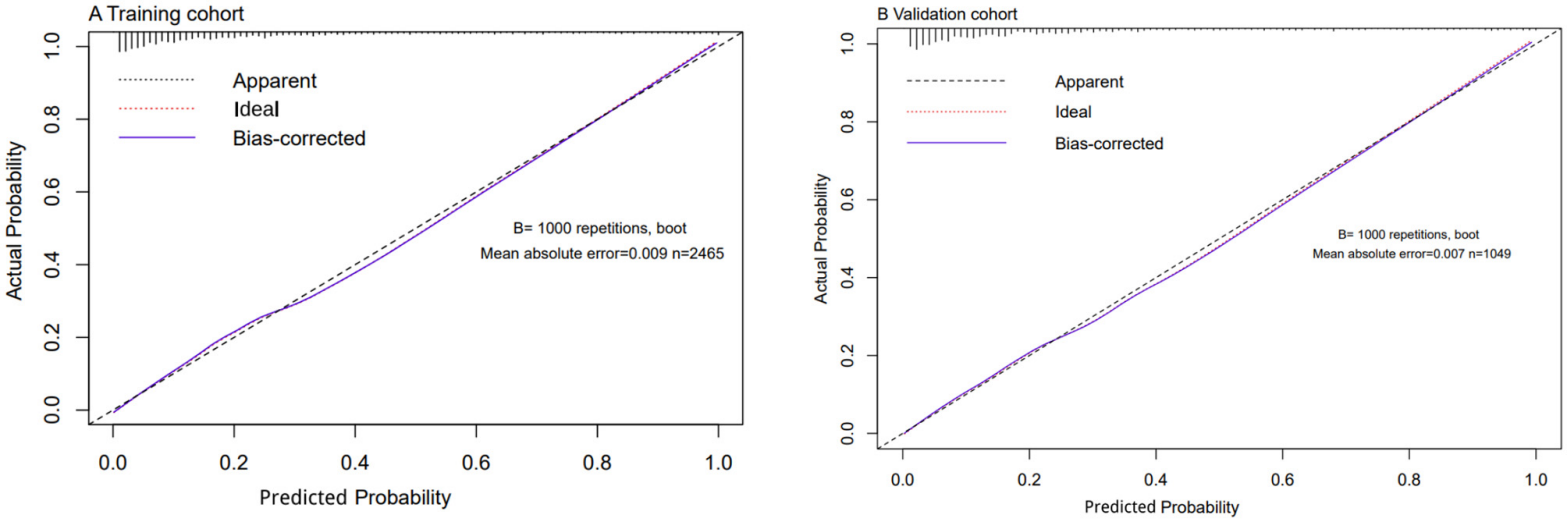
Calibration curves for the nomogram. **(A)** Training cohort. **(B)** Validation cohort.

**Figure 5:**
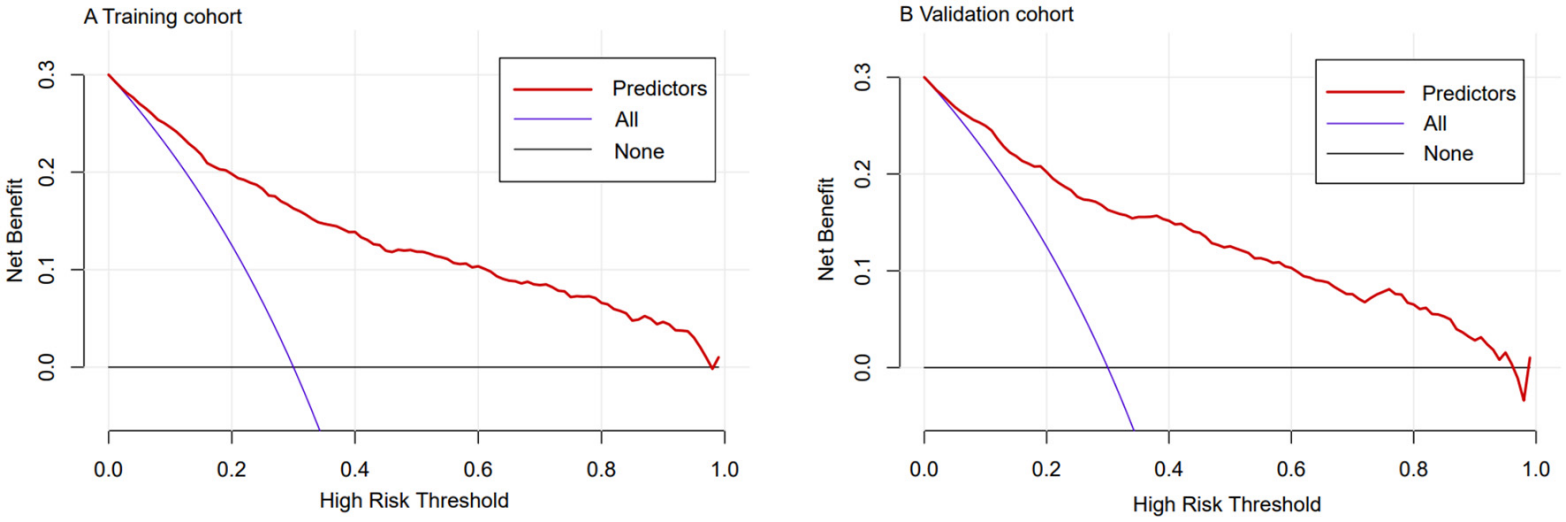
Decision curve analyses demonstrating the net benefit associated with the use of the nomogram for the detection of postpartum pelvic floor muscle weakness (PFMW). **(A)** Training cohort. **(B)** Validation cohort.

Using C-statistics, we evaluated the discriminatory power of the nomogram for primiparous women with PFMW. The C-statistic for the nomogram used to predict PFMW in the training cohort was 0.866 (95% CI 0.850–0.882) (Figure 3). Both the internally cross-validated training cohort and the validation cohort [0.870 (95% CI 0.819–0.903)] had stable C-statistic values (Figure 3). The ability to predict PFMW incidence was compared using the Delong test and receiver operating characteristic (ROC) analysis. The nomogram's C-statistics were clearly superior to those of any independent factors alone (*P* < 0.001 or *P* = 0.008) (Table 3). In the training and validation cohorts, a calibration curve overlapped the ideal line, thus demonstrating good agreement between the actual probabilities and the PFMW probabilities predicted by the nomogram (Figure 4). The positive net benefit associated with using the nomogram to detect PFMW varied from 0.0 to 0.3 in both the training and validation cohorts (Figure 5).

**Table 3:** C statistics for the nomogram and model variables in the training and validation cohorts.

**Variables**	**Training cohort**			**Validation cohort**		
	**C-statistic**	**95% CI**	^*^ ***P***	**C-statistic**	**95% CI**	^*^ ***P***
Nomogram	0.866	0.850–0.882		0.870	0.819–0.903	
Age, year	0.617	0.591–0.634	< 0.001	0.614	0.563–0.672	< 0.001
Weight gain in pregnancy, kg	0.664	0.640–0.687	< 0.001	0.679	0.643–0.715	< 0.001
Mode of delivery	0.549	0.522–0.576	< 0.001	0.556	0.513–0.599	0.008
Second stage of labor, min	0.745	0.723–0.766	< 0.001	0.738	0.703–0.773	< 0.001
Infant weight, g	0.759	0.738–0.780	< 0.001	0.765	0.732–0.797	< 0.001

C statistic, concordance statistic; CI, confidence interval.

^*^The Delong test was used to compare the C statistics.

## Discussion

In this study, we developed and validated a simple nomogram to quantify the risk of PFMW for primiparous women in the early postpartum period. This nomogram, which is based on maternal and obstetrical characteristics, has excellent discriminatory ability, calibration, and net benefit in predicting PFMW. Early rehabilitation is critically important for preventing the progression and persistence of PFD (24–26). Thus, early prediction of PFMW could help clinicians provide professional counseling as well as rehabilitation guidance to the appropriate population.

Some previous clinical studies have assessed the probability of early postpartum PFMW among primiparous women (27–30). However, their findings have been limited due to small sample sizes and the pooling of women who had cesarean sections with those who had vaginal deliveries. Pregnancy and delivery are the most important risk factors for PFD (31, 32), and there are significant differences in baseline factors between cesarean and vaginal deliveries. In the present study, we created a predictive model in a larger cohort on the basis of a detailed collection of obstetrical and especially labor-related factors, which have been well validated. To our knowledge, this is the first predictive model to collect detailed delivery information for predicting the occurrence of postpartum PFMW among primiparous women with vaginal deliveries. Five factors were identified to be predictive of postpartum PFMW in the model, namely, age, weight gain during pregnancy, mode of delivery, duration of the second stage of labor, and infant weight.

PFMW is generally considered strongly associated with maternal age. With increasing maternal age and decreasing estrogen levels, decreased collagen synthesis and reduced tissue elasticity collectively weaken the biomechanical capacity of pelvic floor muscles and connective tissues, thereby increasing their vulnerability to mechanical stress. During pregnancy, the sustained mechanical load from the gravid uterus exerts chronic compressive and stretching forces on these pelvic floor tissues (25). Both Peinado-Molina et al. (31) and Xu et al. (19) reported a significant association between age and PFD when age was treated as a continuous variable. Similarly, we found that the risk of postpartum PFMW increased with each additional year of age (OR 1.156, 95% CI 1.116–1.999). Therefore, more attention should be devoted to the occurrence of postpartum PFMW and the enhancement of pelvic floor muscle training in aging mothers (26).

We found that both gestational weight gain and fetal birth weight were valuable predictors of postpartum PFMW, which is consistent with the findings of previous studies (19, 30, 33). It is well known that weight gain above normal during pregnancy and pre-pregnancy obesity (BMI > 25) increase abdominal pressure and pelvic floor muscle burden, leading to chronic strain and decreased elasticity of muscles and connective tissues. Additionally, there is an increased risk of mechanical injuries during large for gestational age (LGA) delivery, such as overstretching of the pelvic floor muscles and nerve injuries, especially when LGA delivery is combined with prolonged labor or instrumental delivery (30). Nowak et al. (34) reported that high BMI was correlated with excessive gestational weight gain (overweight: OR 3.0, 95% CI 1.84–3.87; obese: OR 2.45, 95% CI 1.1–5.48), and obese women with adequate gestational weight gain had a greater risk of having LGA newborns (OR 5.48, 95% CI 1.15–26.13). Hence, the above three factors have a cumulative superimposed effect on pelvic floor muscle injury, and they themselves can influence each other to some extent. Several studies have shown an increase in the prevalence and severity of PFD with increasing pre-pregnancy BMI compared with normal weight (15, 33). However, there was no association between pre-pregnancy BMI and postpartum PFMW after confounding factors were corrected for in our study, which may have resulted from the combined effects of study population differences, hospital conditions, and postpartum screening methods. Alternatively, weight gain during pregnancy and fetal birth weight may mask the effect of pre-pregnancy BMI on postpartum PFMW to some extent.

To date, the risk of postpartum PFMW for forceps delivery, vacuum delivery, and spontaneous vaginal birth has not been evaluated in large-sample randomized clinical trials. Three studies investigated the associations between postpartum PFMW and delivery mode. In the first cross-sectional study (29), the clinical demographic characteristics and vaginal sEMG data of 1,259 primiparous women at 6–8 weeks after birth were collected. The magnitude of fast and sustained contractions on sEMG in the forceps delivery group was significantly lower than that in the spontaneous vaginal birth group (*P* < 0.002, *P* < 0.001), and forceps delivery significantly inversely influenced PFM activity (fast contractions: OR 1.779, 95% CI 1.103–2.869; sustained contractions: OR 2.197, 95% CI 1.378–3.503). In the second study (28), 247 (85.5%) patients underwent vacuum-assisted delivery, and 42 (14.5%) patients underwent forceps delivery. The prevalence rates of levator ani muscle injury after vacuum and forceps delivery were 16.6% (41/247) (95% CI 12.0–21.2) and 40.5% (17/42) (95% CI 25.6–55.4), respectively (*P* = 0.001). Forceps delivery was identified as a risk factor for levator ani muscle injury, with an OR of 3.537 (95% CI 1.7–7.3). In the third study (35), among 45 participants in the forceps group and 28 participants in the vacuum group, the prevalence of levator ani muscle avulsion was significantly greater after forceps than after vacuum delivery [22/45 (49%) vs. 5/28 (18%), *P* = 0.012, prevalence ratio 2.74, 95% CI 1.17–6.40, OR 4.40, 95% CI 1.42–13.62]. However, the above two studies both lacked a spontaneous vaginal delivery group as a baseline control group, limiting the power. PFMW can be caused by levator *ani muscle* injuries and extensive levator hiatus (36). Thus, our findings were largely consistent with the conclusions of the above three studies, with the advantage of further quantifying the effect of vaginal delivery mode on postpartum PFMW, with a C-statistic of 0.549 (95% CI 0.522–0.576, *P* < 0.001). Forceps delivery was an independent risk factor (OR 1.904, 95% CI 1.336–2.714) and therefore was included in the nomogram model, whereas vacuum delivery was not.

The prolonged second stage of labor was shown to be another important predictor of postpartum PFMW in our study and previous studies (27, 36). During labor, the levator ani muscle and birth canal tissues must stretch to more than three times their original length. Continuous breath holding and exertion cause the pelvic floor muscle fibers and connective tissues to stretch repeatedly beyond their elastic limits, triggering microscopic tears. Prolonged compression of the pelvic floor nerves (e.g., pubic nerves) by the fetal head leads to nerve conduction dysfunction and weakens muscle contraction (36). Therefore, both the increased number of impairments and the prolonged duration of stress on the pelvic floor structure are associated with the onset of postpartum PFMW.

The current study has several limitations. First, we focused only on the detection of vaginal surface EMG signals, and further diagnoses of PFMW are lacking. Second, since third- and fourth-degree perineal lacerations are rare, analyzing them together with episiotomy may affect statistical validity. Third, several potential factors related to demographics and the postpartum recovery phase (e.g., economic level, postpartum breastfeeding, pelvic floor function exercise, etc.) were not considered. More potential indicators combined with clinical characteristics should be investigated to build a more accurate prediction model for postpartum PFMW. Finally, almost all the participants in this study were residents of Shanghai, an economically developed region of China. Therefore, further multicenter clinical trials are needed to establish a more general predictive model.

The novel clinical prediction model in the present study has practical implications since it is simple to adopt, shows good discrimination, and demonstrates good calibration for predicting the occurrence of early postpartum PFMW among primiparous women with vaginal delivery. We plan to integrate this predictive model into the routine 42-day postpartum examination at our hospital, enabling refined management of pelvic floor muscle function for postpartum women. By categorizing individuals into different risk groups for stratified management. The nomogram might help primiparous women with vaginal delivery benefit from early detection and rehabilitation of PFMW, thus facilitating the management of pelvic floor disorders. However, the benefits remain to be explored in prospective trials.

## Conclusion

We created a nomogram that can be utilized to quantify the risk of postpartum PFMW for primiparous women with vaginal delivery. The model developed herein might contribute to the early identification of postpartum PFMW, thereby providing evidence-based strategies for preventing postpartum and long-term PFD.

## Data Availability

The raw data supporting the conclusions of this article will be made available by the authors, without undue reservation.
