# Correction: Establishment and validation of a clinical prediction model for predicting early postpartum pelvic floor muscle weakness among primiparous women after vaginal delivery: a retrospective study

**DOI:** 10.3389/fmed.2025.1750052

**Published:** 2025-12-03

**Authors:** Huan Dong, Xiaolei Chi, Ye Liu, Wenjuan Liu, Xinliang Chen, Xianjing Wang, Ping Liu

**Affiliations:** 1 The International Peace Maternity and Child Health Hospital, School of Medicine, Shanghai Jiao Tong University, Shanghai, China; 2 Shanghai Key Laboratory of Embryo Original Diseases, Shanghai, China

**Keywords:** primiparous women, vaginal delivery, pelvic floor muscle weakness (PFMW), clinical prediction model, early postpartum period

The funder Sailing Special Project of the Shanghai Rising-Star Program (No. 23YF1451900) and the National Natural Science Foundation of China (8237060413) was erroneously omitted.

The corrected **Funding** statement is:

“The author(s) declare that financial support was received for the research and/or publication of this article. This research was supported by the funder Sailing Special Project of the Shanghai Rising-Star Program (No. 23YF1451900) and the National Natural Science Foundation of China (8237060413).”

The original version of this article has been updated.

